# Mathematical and Machine Learning Models of Renal Cell Carcinoma: A Review

**DOI:** 10.3390/bioengineering10111320

**Published:** 2023-11-16

**Authors:** Dilruba Sofia, Qilu Zhou, Leili Shahriyari

**Affiliations:** Department of Mathematics and Statistics, University of Massachusetts Amherst, Amherst, MA 01003, USA; dsofia@umass.edu (D.S.); qiluzhou@umass.edu (Q.Z.)

**Keywords:** mathematical modeling, neural network, machine learning, differential equation, sunitinib, checkpoint inhibitors, gene signature

## Abstract

This review explores the multifaceted landscape of renal cell carcinoma (RCC) by delving into both mechanistic and machine learning models. While machine learning models leverage patients’ gene expression and clinical data through a variety of techniques to predict patients’ outcomes, mechanistic models focus on investigating cells’ and molecules’ interactions within RCC tumors. These interactions are notably centered around immune cells, cytokines, tumor cells, and the development of lung metastases. The insights gained from both machine learning and mechanistic models encompass critical aspects such as signature gene identification, sensitive interactions in the tumors’ microenvironments, metastasis development in other organs, and the assessment of survival probabilities. By reviewing the models of RCC, this study aims to shed light on opportunities for the integration of machine learning and mechanistic modeling approaches for treatment optimization and the identification of specific targets, all of which are essential for enhancing patient outcomes.

## 1. Introduction

Renal cell carcinoma (RCC), the predominant form of kidney cancer that originates from the kidney tubules, poses a formidable global public health challenge and is associated with 80% to 90% of kidney cancer [1,2,3,4,5]. In 2020, there were over 430,000 new cases of RCC, with around 179,368 deaths worldwide, with the highest incidence recorded in the USA [6]. It constitutes approximately 2–3% of adult tumors and 7% of childhood tumors, making RCC’s impact profound [5,7,8,9]. Although it accounts for 2–3% of all cancer types, the number of new cases is overall increasing [10]. The projected surge in new RCC cases and associated deaths in the United States alone is expected to reach 81,800 and 14,890, respectively [11,12]. Most diagnosis of RCC happens via chance through imaging, indicating that RCC is more prevalent than the diagnosed cases [13]. Often, the patient’s survival depends on the stage of the tumor and whether they have metastases or not [13]. Furthermore, only 12% of patients with metastatic RCC have 5-year survival prognoses [13]. The growing cases and poor survival highlight the urgent need for a better understanding of RCC and calls for innovative techniques such as machine learning or mathematical modeling to transform approaches to diagnoses, treatment, and survival outcome of RCC patients.

Genetic mutations play a central role in the development of RCC. Approximately 2–3% of RCC cases have a familial link, which doubles the risk of RCC in first-degree relatives of patients and is attributed to hereditary gene mutations [5]. Notably, the Von Hippel–Lindau (VHL) gene, implicated in familial RCC, orchestrates hypoxia-inducible factor (HIF) pathways that significantly affect angiogenesis and cell survival [2,14,15,16]. In the context of hereditary papillary RCC, the c-met proto-oncogene encodes a growth factor receptor that promotes tumor growth. Additionally, the fumarate hydratase gene contributes to the aggressive growth of tumors in familial Leiomyomatosis RCC, particularly affecting women with a history of early hysterectomy and associated with Type 2 papillary RCC [5]. Another frequently mutated gene, polybromo-1 (PBRM-1), plays a critical role in chromatin remodeling and tumor suppressor function [1]. These genetic abnormalities collectively contribute to the genetic diversity observed in RCC, resulting in variations in tumor behavior, treatment responsiveness, and clinical outcomes [14].

Oncological treatment for RCC has seen a revolution in its treatment landscape in the last two decades, moving from a 5–10% treatment response of metastatic patients pre-2004 to 30–40% patients responding to the targeted therapeutic agents between 2005 and 2014 [17]. With the use of immune checkpoint inhibitors (ICIs), the response rate for metastatic RCC patients rises to 60%, along with a higher survival rate [17]. The treatment landscape for RCC spans a spectrum of modalities in these time periods. Surgical resection remains pivotal for localized tumors, while advanced cases necessitate targeted therapies like immunotherapy and molecularly targeted agents [1,4,18,19]. Immunotherapies, including immune checkpoint inhibitors like nivolumab and pembrolizumab, seek to mobilize the immune system against cancer cells by disrupting evasion mechanisms [20,21,22,23,24,25,26,27,28,29,30,31,32,33,34,35,36,37,38,39,40,41]. Molecularly targeted agents, such as sunitinib and pazopanib, leverage specific genetic alterations to inhibit angiogenesis and tumor growth pathways [23,30,38,42,43,44,45,46,47,48]. These recent treatment options can complement the treatment landscape of RCC using complementary molecular mechanism based on the studies of biology, pathology, and genomics of renal carcinoma and can ultimately start the diamond age of precision treatment where RCC medicine can be tailored to specific patients based on biological and pathological circumstances [17].

Recognizing the strains imposed by clinical trials on patients’ well-being, researchers have diligently sought optimized treatments and an expanded understanding of RCC through diverse models, including in vitro or in vivo models such as mice, cell lines, machine learning, and mathematical frameworks [49,50,51,52,53,54,55,56,57,58,59,60,61]. Moreover, researchers have attempted to predict patient outcomes and improve treatment options for patients using various models and findings [21,36,40,45,47,62,63,64,65,66,67,68,69,70,71,72,73].

Machine learning models can provide valuable insights into patient datasets, whether they are discrete or based on black-box models [60,61]. For instance, these models excel at prognostic analysis and the identification of potential biomarker genes crucial to RCC by analyzing patient clinical and gene expression data. For instance, Terrematte et al. identified a 13-gene signature through the integration of eight machine learning models [74]. Similarly, Zhan et al. discovered a five-gene signature using a random forest algorithm [60]. Satter et al. utilized an unsupervised learning approach, employing Uniform Manifold Approximation and Projection (UMAP) as well as density UMAP to distinguish tumor types, further selecting the Chromophobe–Oncocytoma gene signature through gene set enrichment analysis (GSEA) [75].

Additionally, machine learning models can analyze the risk factors associated with specific diseases based on patient information. Yin et al. [61] integrated Convolutional Neural Network (CNN) models with Cox regression to identify potential prognostic biomarkers for overall survival. Furthermore, machine learning models excel in classifying and predicting renal cancer, whether using medical images or genomics data. CNNs are tailor-made for processing data represented as multidimensional arrays, enabling them to capture spatial relationships within image data [76]. Han et al. employed CNNs to classify renal cell subtypes by feeding them three-phase CT images [77]. CNNs have also been applied effectively in analyzing genomics data and making predictions. Alternatively, Recurrent Neural Networks (RNNs) can process sequential data, such as time-course gene expression data. However, the scarcity of time-series gene expression data can limit the application of RNNs for cancer predictions. As a result, some studies prefer CNNs over RNN methods, as they can be seamlessly integrated with other statistical or mechanistic models and are more amenable to parallel computing [78].

Conversely, mechanistic models offer a comprehensive elucidation of the underlying mechanistic processes governing the pathophysiology of cancer progression [79,80,81,82,83,84,85]. These models also serve as invaluable tools for exploring novel immune pathways aimed at eradicating tumors, establishing precise intervention timing and addressing various aspects of disease management [24,86,87]. In cases where the objective is to mitigate cancer growth in a patient, the model can be parameterized using patient-specific data to identify pivotal factors or agents capable of diminishing tumor size or achieving complete tumor regression. In instances where a complete cure is not feasible, simulations can be employed to ascertain the optimal equilibrium for the patient to sustain a manageable disease burden [88,89].

Several mechanistic mathematical models have been developed to investigate RCC progression and response to treatments [49,50,59,90,91,92,93,94,95]. For example, De Pillis et al. developed a model that primarily focuses on determining the optimal Sunitinib dosage [49], while Sofia’s model concentrates on exploring the role of immune cells in cancer progression [93]. For modeling RCC metastases, Hanin et al. sought to elucidate the temporal progression from tumor removal to metastatic development [50], while Barachart et al. directed their focus toward investigating the potential merging of metastatic foci within the pulmonary region [94]. It is noteworthy that insights acquired from these mechanistic models collectively contribute to the refinement of comprehensive strategies for the management of RCC patients.

We provide a review of some more recent mechanistic and machine learning models for both primary and metastatic RCC, with a specific focus on the works of Zhan et al. [60], Yin et al. [61], and Su et al. [4], complemented by prominent mechanistic models by De Pillis et al. [49], Sofia et al. [93], Hanin et al. [50], and Barachart et al. [94]. These models have emerged as tools for unraveling the dynamics of RCC and optimizing treatments. While the landscape of mathematical and machine learning models is ever-evolving, the practical implications of these findings hold promise for real-world applications. Our goal with this review is to collectively elaborate on the techniques and findings of the aforementioned models.

## 2. Machine Learning Models

**Figure 1:**
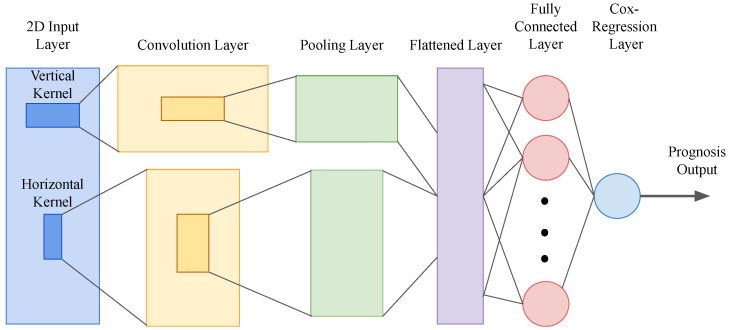
Structure of the CNN-Cox model by Yin et al. [61]. Two kernels are used in the CNN-Cox model. The horizontal kernel slides horizontally with the size of a column, and the vertical kernel slides vertically with the size of a row. These two convolutional kernels are applied to the 2D input matrix to extract local features. Then, the output is passed to a max-pooling layer, a flattened layer, a fully connected layer, and an output Cox-regression layer.

The neural network model that Yin et al. created employs a convolutional neural network (CNN) with a Cox model to analyze the prognosis of multiple cancer types, including RCC [61] (see Figure 1 for the model structure). They used The Cancer Genome Atlas (TCGA) pan-cancer RNA-seq dataset from UCSC Xena platform [96,97] to train the model. To mitigate the overfitting problem caused by high-dimensional genomic data, they applied cascaded Wx(CWx) feature selection before implementing the CNN-Cox model [98]. The model can be implemented as either a 1D-CNNCox model based on the 1D-CNN framework or a CNN-Cox model combining the 2D-Hybrid-CNN framework with the CoxPH model. In the 1D-CNNCox model, the input consists of one patient’s gene expression data represented as a 1D vector. The CNN-Cox model takes 2D data as input, where the gene expression data of multiple patients form a two-dimensional matrix, with patients as columns and gene expression as rows. The difference is that the CNN-Cox model has two sets of features from the rows and columns, in contrast to a single set of features in the convolution and pooling layers before flattening. After flattening the layers, they found a fully connected layer of nodes before applying the Cox-Regression analysis to determine the survival outcome of the patients.

Yin et al.’s findings revealed that the CNN-Cox method outperformed earlier survival analysis models. Notably, they identified four specific genes, ADH6, ALDH3A2, BBOX1, and GATM, as hub genes that significantly interact with many of the other genes in each patient, revealing that these genes might be good targets to treat RCC patients [61].

**Figure 2:**
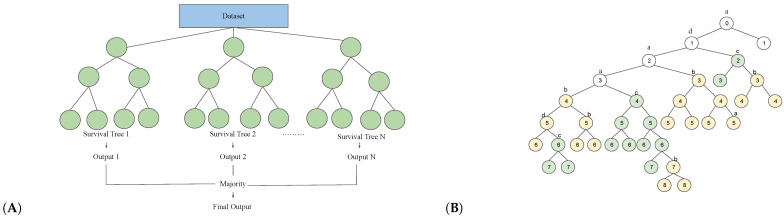
Illustration of the RSF-VH algorithm. (**A**) shows the structure of the RSF-VH algorithm. N bootstrap samples and survival trees are drawn and grown from the dataset. At each node, a randomly selected subset of genes with minimal depths smaller than the mean of estimated minimal depth of the forest is considered to split the branch. This process is repeated until a stopping criterion is met. (**B**) illustrates the minimal depth of genes b and c in a survival tree. a, b, c, and d are four different randomly selected genes, and the numbers inside each node indicate the depth of the tree. Yellow and green colored points are maximal subtrees for genes b and c, respectively. And the minimal depth for gene b is 3 and for gene c is 2.

Zhan et al. analyzed survival prediction using KIRC patient sample RNA expression data from next-generation sequencing and clinical information from the TCGA dataset [60,97]. They conducted a univariable Cox regression analysis with a significance level of 0.001 on the log2 transformed gene expression data to identify genes that are potentially correlated with patients’ survival. Then, they used the random survival forests–variable hunting (RSF-VH) algorithm with the default settings to select the 100 most important genes [99]. In RSF-VH, the survival predictiveness of a gene is measured using the minimal depth of the maximal subtree for that given gene. Genes with smaller minimal depth contribute more effectively to the survival outcome (See Figure 2). To further reduce the dimensions of genes, a five-gene model was determined by performing a Cox proportional-hazard regression analysis with an enumeration algorithm.

As a result, Zhan et al. found five gene markers, CKAP4, ISPD, MAN2A2, OTOF, and SLC40A1, that play crucial role in RCC patient survival [60]. The idea of these genes as potential biomarkers of RCC was supported by several other studies, including Sun et al. [100], Miller et al. [101], Cox et al. [102], and Zhang et al. [103].

On the other hand, Su et al. [4] approached their analysis differently. Instead of directly working with genomics data like Yin et al. and Zhan et al., Su et al. focused on the immune aspect of clear cell RCC using the TCGA gene expression profiles from the primary tumors obtained from UCSC Xena [96,104]. Although their analysis was rooted in gene expression and clinical data of patients [4], their methodology stood apart. They initially generated an immune cell frequency matrix for each patient using a digital cytometry technique known as CIBERSORTx, employing the LM22 signature matrix [105,106]. Subsequently, Su et al. applied K-means clustering methods to the cell frequencies, unveiling distinct immune patterns within ccRCC tumors. The results disclosed the presence of four distinct groups of ccRCC tumors, each characterized by a unique immune composition. Furthermore, their statistical analysis brought out significant findings. They demonstrated that higher-grade and -stage ccRCC tumors exhibited a higher proportion of CD8+ T-cells but lower proportions of mast cells and monocytes. Importantly, a negative correlation was identified between the number of macrophages and CD8+ T-cells, while a significantly positive correlation emerged between the expression levels of PDCD1 and INFG with the percentage of CD8+ T-cells in ccRCC tumors.

One of the main concerns in working with machine learning models involves the overfitting problem associated with high-dimensional genomics data. Yin et al. addressed this issue by implementing CWx feature selection to rank genes based on their discriminating power. In a similar vein, Zhan et al. utilized the RSF-VH algorithm to select genes with the largest importance value. Additionally, Su et al. employed the CIBERSORTx algorithm to downscale the gene expression data to frequencies of 22 immune cells. Regarding statistical methods, both Yin et al. and Zhan et al. integrated machine learning models with Cox regression.

## 3. Mechanistic Models

In this section, we begin by discussing two models for RCC that are based on ordinary differential equations (ODEs) and were developed by De Pillis et al. [49] and Sofia et al. [93]. Subsequently, we discuss two additional models concerning RCC metastasis, which were formulated by Hanin et al. [50] and Barachart et al. [94].

**Figure 3:**
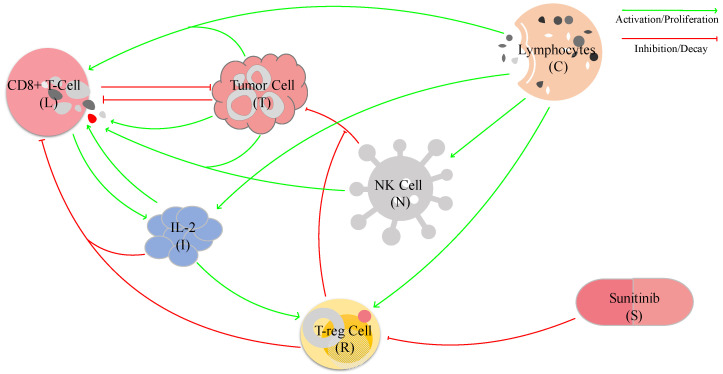
The network of interactions between immune and tumor cells including IL-2 and Sunitinib modeled by De Pillis et al. [49]. CD8+ T-cell and NK cell inhibit tumor cells. But NK cells’ action against tumor cells is obstructed by T-reg cells. Tumor cells in turn inhibit CD8+ T-cells and NK cells. Besides tumor cells, T-reg cells along with IL-2 inhibit CD8+ T-cells. CD8+T cells can be recruited by the debris from tumor cells generated by NK cells, and the immune system can be stimulated by the presence of the tumor to create more CD8+T cells. Lymphocytes promote all the immune cells in the model, while IL-2 promotes all other immune cells except lymphocytes. Last but not least, Sunitinib inhibits T-reg cells that have an indirect effect on the tumor microenvironment.

**Figure 4:**
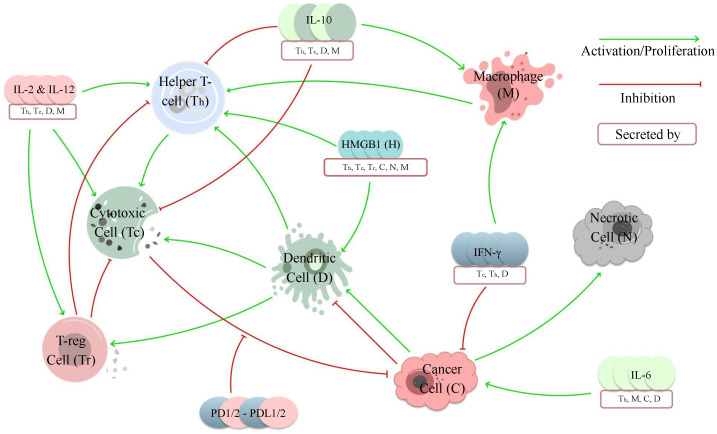
The network of interactions is modeled by Sofia et al. [93]. In this model, CD8+ T-cells, NK cells (cytotoxic cells), and IFN-γ inhibit cancer cells. But when the programmed cell death ligand binds with the programmed cell death proteins, CD8+ T-cells’ death rates of cancer cells are reduced. Cytokines such as IL-2 and IL-12 promote cytotoxic, helper, and regulatory T-cells. However, IL-10 inhibits cytotoxic cells and helper T-cells and promotes macrophages. IFN-γ also promotes macrophages. On the other hand, macrophages promote helper T-cells. Dendritic cells promote T-reg, helper, and cytotoxic cells. Cancer cells can promote and inhibit dendritic cells, and IL-6 can increase cancer cells’ proliferation. HMGB1, which is mainly produced by cancer and necrotic cells, promotes helper T-cells and dendritic cells. In addition, cancer cells contribute to the necrotic core due to the fast growth and death of cancer cells.

Both De Pillis et al. and Sofia et al. employed a deterministic model and used mostly mass action law to model the rate of changes in the number of cells $X_{i}$ in the tumor microenvironment through the equations like the following:

(1) $$\begin{array}{c} \frac{dX_{i}}{dt}=\sum_{j}\lambda_{ji}Y_{j}X_{i}-\sum_{k}\delta_{ki}Y_{k}X_{i} \end{array}$$

where $Y_{j}$ values are the variables that are affecting $X_{i}$, $\lambda_{ji}$ values are the growth or activation rates of $X_{i}$ by the variable $Y_{j}$, and $\delta_{ki}$ values are the decay or death rates of $X_{i}$ caused by $Y_{k}$. To bound cancer growth, both studies have used a logistic model, and to describe some of the decay rates, both used a hill-type function. However, each of these models is composed of a different set of variables and parameters as they model different networks of interactions (see Figure 3 and Figure 4). Further, De Pillis et al. also employed the Michaelis–Menten law and exponential decay to describe some of the interactions between cells and cytokines.

The mechanistic models developed by De Pillis et al. [49] and Sofia et al. [93] both focus on modeling the interactions between immune and cancer cells within the tumor microenvironment. However, they consider distinct sets of interactions, as illustrated in Figure 3 and Figure 4. De Pillis et al. concentrated on optimizing Sunitinib—a drug that can suppress the activity of regulatory T cells’ dosage options based on the specific characteristics of patients’ tumor microenvironments, while Sofia et al. explored the impact of the immune landscape of tumors on their progression.

De Pillis et al. integrated quantitative data on various cell types and cytokine concentrations per liter of blood into their model, relying on prior biological evidence that established the ratios of these cell types and cytokine concentrations. In contrast, Sofia et al. employed TCGA data to infer cytokine concentrations and the number of cells in the tumors. The parameter estimation approaches also differed. De Pillis et al. derived most of their model parameters from a combination of earlier studies, while Sofia et al. assumed that large tumors are at the steady state and used TCGA data to estimate most of the parameters [49,93]. For precision medicine, Sofia et al. estimated model parameters separately for each patient group exhibiting specific immune patterns identified by Su et al. [4], enabling a tailored investigation of tumor progression for each patient cluster.

Both studies conducted sensitivity analyses, but the methodologies varied. De Pillis et al. employed local sensitivity analysis, while Sofia et al. used global sensitivity analysis [49,93]. Sofia’s team identified key pathways that could potentially slow down cancer cell growth, emphasizing the roles of dendritic cells and the secretion rate of IL-6 as promising targets for inhibiting or decelerating cancer growth [93]. On the other hand, De Pillis and her collaborators simulated the effects of Sunitinib on virtual patients. The findings show that, in a simulated population of 100, standard Sunitinib treatments improved tumor control in around 30% to 40% of patients, aligning with clinical studies. Simulating treatments with a double dose increased the response rate to about 15%, but approximately 45% of patients still did not respond.

For modeling RCC’s metastases, Hanin et al. introduced a probabilistic model aimed at comprehending the emergence of lung metastases from primary RCC, aiding in the scheduling of treatments based on metastasis growth rates [50]. Their stochastic framework introduced an element of uncertainty, mirroring the intrinsic variability in metastatic progression.

The metastasis model by Hanin et al. focuses on fitting the sizes of lung metastases in four patients to a probability density function, which is dependent on the growth of the primary tumor [50]. They assumed that metastases are shed off the primary tumor according to a Poisson process whose rate is μ(t) = α$\Phi^{\theta }\left(t\right)$, where Φ(t) is the size of the primary tumor at time *t*, and θ and α are the parameters of the model. They assumed two different growth models for the primary tumor, the exponential model and the Gompertz growth model [50]. In both of these scenarios, if the metastases’ latency times are negligible, then the probability density function becomes an instantaneous seeding model, and if the latency times infinitesimally long, then the probability density function becomes a long-latency model.

Hanin et al.’s model suggests that metastatic cells likely originate in the lungs immediately, indicating that the time it takes for metastatic cells to travel from the kidney tumor to the lungs is negligible compared to the period of metastatic growth. Moreover, the early detection and surgical treatment of the primary tumor would not have prevented metastatic spread in the analyzed patients. They also found that the primary kidney tumor had varying degrees of suppression on metastatic growth, and this suppression was not directly proportional to tumor size [50]. Importantly, the number and volume of lung metastases were not directly proportional to the size of the primary tumor. Additionally, the rate of metastasis shedding depended on the parameter θ, which indicated the presence of metastasis-producing cells within the primary tumor. Furthermore, the number of detected lung metastases did not correlate with θ, suggesting patient-specific factors and detection thresholds. Their findings suggest an aggressive cancer phenotype in patients with rapid metastatic growth.

In order to enhance our understanding of how metastases form, Barachart and colleagues conducted experiments on Balb/c mice by injecting RENCA cells into their kidneys and observing the development of metastases in the lungs [94]. Initially, they attempted to correlate their findings with the green fluorescent protein (GFP) signals of mice’s metastases, assuming that metastatic tumors either grow independently of each other or do not exhibit spatial interactions [94,95]. Following this assumption and considering the primary tumor’s adherence to Gompertz growth, they developed a mathematical model using a transport partial differential equation (PDE) to describe the density of metastatic tumor sizes over time [94,95]. However, the metastatic burden predicted by this transport equation did not align with the observed GFP signaling data of metastatic burden in the mice [94]. Consequently, they devised an advection-type system of PDE that characterizes the density of both healthy and tumor cells, accounting for the possibility that pressure may obstruct tumor growth as well. Their model ultimately suggested that metastases in close proximity can indeed merge, and this modeling approach is consistent with the metastatic burden they identified through MRI and GFP signaling in their mice experiment [94].

## 4. Discussion

This review covers some machine learning and mechanistic models of RCC. The machine learning studies reviewed here collectively emphasize the pivotal role of genetic factors in the development and prognosis of RCC. The identification of specific genes, such as VHL and PBRM-1, highlights the genetic heterogeneity of RCC, leading to variations in clinical behavior and treatment responses. In particular, Zhan et al. [60] found ISPD and CKAP4 to be key genes in predicting RCC, and these results are supported by clinical and experimental observations [100,101]. Miller et al. [101] found that reduced levels of ISPD increased the odds of the grade and stage of the tumor, as well as patient mortality [101]. Moreover, Sun et al. [100] noted that the upregulation of CKAP4 has worse characteristics, including shorter overall survival and progression-free period. Machine learning models have also been instrumental in identifying genes linked to a favorable prognosis in RCC. For instance, GATM has been pinpointed as a potential tumor suppressor [107]. Beyond specific genes, immune clusters and cells are implicated in poor prognosis. Su et al.’s research [4], revealed that Cluster (CD4 < MΦ < CD8) has the highest grade and stage of tumors. It also found that tumors of higher grade and stage tend to have a higher proportion of CD8+ T-cells and lower percentages of mast cells and monocytes. These histological features not only provide insights into the disease’s etiology but also offer potential targets for therapeutic interventions. Future research in this area could focus on further elucidating the molecular mechanisms behind these genetic and phenotypic alterations and their precise impact on RCC progression. It is important to note that compared with mechanistic models, machine learning models such as the studies by Yin et al. [61] and Zhan et al. [60] tend to be less explainable because they are black box models. Nevertheless, they offer greater flexibility when it comes to integration with other statistical survival models.

The mechanistic models discussed in this paper, whether deterministic or probabilistic, offer valuable tools for understanding RCC dynamics. Both De Pillis et al.’s [49] and Sofia et al.’s [93] models show that patient-specific immune strength is a critical factor in explaining the differences in tumor progression and treatment response. The sensitivity analyses of these models reveal possible key factors in tumor progression. By systematically varying these sensitive factors within the model, researchers can gain insights into how RCC responds to different perturbations and interventions. For instance, Sofia et al. [93], by performing sensitivity analysis, found that the decrease in IL-6 production and decrease in dendritic cells’ decay rates can slow down cancer growth as IL-6 directly promotes cancer growth and dendritic cells indirectly suppress cancer growth via helper T-cell and CD8+ T-cell [36,93,108,109,110,111]. Therefore, if a RCC patient has increased the production of IL-6 or increased the decay of dendritic cells, then this patient might have a poor prognosis. This information is crucial for optimizing treatment plans, identifying potential therapeutic targets, and developing a more comprehensive understanding of the disease’s behavior over time.

In the field of mechanistic modeling, applications of ODEs, PDEs, and probabilistic models in cancer modeling are widespread. In addition to these models, there are fractional differential equation (FDE) models, which have been applied to modeling cancer and infectious diseases [112,113,114,115]. The fractional order in the FDE models would allow flexibility in capturing complex behaviors in cancer dynamics, so it would also be a valuable tool to be utilized for understanding the complexity of RCC.Ultimately, these mechanistic models enhance our ability to make informed decisions in the field of RCC research and clinical practice.

Although the mechanistic models convey important findings, it is crucial to consider that these models cannot capture the complete biological complexity that each patient exhibits. Applications of these models when deciding treatment approaches for an RCC patient may become reduced or even enhanced based on each patient’s health. Furthermore, these models make some assumptions to avoid unnecessary complexity that might reduce the accuracy of their predictions. For example, Sofia et al. assumed that large tumors are at a steady state to be able to estimate the parameters of their models using the available data. Hanin et al. also assumed that the sizes of the metastases are uniformly distributed. However, they did not consider that metastatic lesions in the lung could merge together, as Barachart et al. observed in their experiments and model [50,94].

Undoubtedly, one of the most pressing challenges facing researchers in the fields of mathematical modeling and machine learning in cancer studies is the acute shortage of comprehensive data. This deficit is most pronounced in the context of time-course patient data, which are crucial for the accurate parameterization of models and the rigorous validation of their outcomes. The complexity of cancer’s multifaceted nature demands a wealth of longitudinal patient data, yet obtaining such data remains a significant hurdle in the advancement of our understanding of the disease and the development of effective predictive models.

Moving forward, it is essential to continue advancing our understanding of RCC through interdisciplinary research. Integrating genetic profiling, immunological insights, and mathematical modeling will contribute to the development of innovative diagnostic methods and treatment strategies. For example, genetic and immune biomarker findings from machine learning methods can be reinforced into mechanistic models. This can be achieved by incorporating additional variables and interactions related to these biomarkers into existing frameworks like those developed by De Pillis et al. [49] and Sofia et al. [93] Such an approach allows for a more profound understanding of how these biomarkers influence tumor growth, either promoting or inhibiting it. Moreover, the accuracy and effectiveness of these enhanced mechanistic models can be further validated using machine learning techniques. For example, to optimize the parameter settings of the mechanistic models by Barachart et al. [94] and Hanin et al. [50], one could employ machine learning methods, utilizing their datasets. This synergy between machine learning and mechanistic modeling opens new avenues for deeper insights and more effective cancer treatment strategies. Moreover, the translation of research findings into clinical practice remains a key challenge. Researchers should work closely with clinicians to validate these findings in patient cohorts and facilitate the development of more effective therapeutic approaches for RCC.

In conclusion, the studies reviewed in this paper collectively provide a multifaceted perspective on RCC. Despite the limitations of the models, they highlight the complex interplay of genetic, immunological, and mathematical factors in the disease’s progression and offer avenues for improving patient care. By further exploring these findings and fostering collaboration between researchers and clinicians, we can strive to enhance our ability to diagnose, treat, and ultimately prevent the devastating impact of RCC. 

## Data Availability

In this study, no new data were created.

## Abbreviations

The following abbreviations are used in this manuscript:

RCC	Renal Cell Carcinoma
GSEA	Gene Set Enrichment Analysis
TCGA	The Cancer Genome Atlas
VHL	Von Hippel–Lindau
HIF	Hypoxia-Inducible Factor
ODE	Ordinary Differential Equation
GFP	Green Fluorescent Protein
MRI	Magnetic Resonance Imaging
PDE	Partial Differential Equation
UMAP	Uniform Manifold Approximation and Projection
CNN	Convolutional Neural Network
RNN	Recurrent Neural Network
RSF-VH	Random Survival Forests-Variable Hunting
FDE	Fractional Differential Equation
